# The expectancy of threat and peritraumatic dissociation

**DOI:** 10.3402/ejpt.v4i0.21426

**Published:** 2013-12-13

**Authors:** Pamela McDonald, Richard A. Bryant, Derrick Silove, Mark Creamer, Meaghan O'Donnell, Alexander C. McFarlane

**Affiliations:** 1 School of Psychology, University of New South Wales, Kensington, New South Wales, Australia; 2 Australian Centre for Posttraumatic Mental Health, University of Melbourne, Melbourne, Victoria, Australia; 3 Department of Psychiatry, University of Adelaide, Adelaide, South Australia, Australia

**Keywords:** dissociation, peritraumatic, Derealization, trauma

## Abstract

**Background:**

Peritraumatic dissociation is one of the most critical acute responses to a traumatic experience, partly because it predicts subsequent posttraumatic stress disorder. Despite this, there is little understanding about the factors that influence peritraumatic dissociation. This study investigated the extent to which peritraumatic dissociation is predicted by the amount of perceived warning that participants had of the impact of the trauma.

**Method:**

Randomized eligible admissions to four major trauma hospitals (*N*=243) were assessed during hospital admission with the Peritraumatic Dissociation Experiences Questionnaire (PDEQ) and the perceived warning that participants had before the trauma impact occurred.

**Results:**

Whereas female gender predicted both Awareness and Derealization subscale scores on the PDEQ, perceived warning also predicted scores on the Derealization subscale.

**Conclusions:**

This finding suggests that the degree of anticipated threat may contribute to peritraumatic dissociation.

Peritraumatic dissociation potentially involves disturbed awareness, impaired memory, or altered perceptions during and immediately after a traumatic experience (Cardeña & Spiegel, 1993). These responses are very common in the immediate aftermath of trauma, with common reports of emotional numbing, reduction in awareness of one's surroundings, depersonalization, and amnesia (Cardeña & Spiegel, 1993; Feinstein, 1989). One of the major reasons much attention has focused on peritraumatic dissociation has been the strong relationship it has with subsequent posttraumatic stress disorder (PTSD). Numerous studies have reported that peritraumatic dissociation is a strong predictor of PTSD (Ehlers, Mayou, & Bryant, 1998; Koopman, Classen, & Spiegel, 1994; Murray, Ehlers, & Mayou, 2002; Shalev, Freedman, Peri, Brandes, & Sahar, 1997), although the relationship appears to be complex (Breh & Seidler, 2007; Velden et al., 2006). It was largely on the basis of this evidence that DSM-IV defined the diagnosis of acute stress disorder, which placed considerable emphasis on dissociative symptoms (Harvey & Bryant, 2002).

There is limited understanding about the factors that lead to peritraumatic dissociation (Bryant, 2007). Most models of peritraumatic dissociation build on the historical notion derived from the work of Janet (1907) that dissociation allows traumatic memories to be split from awareness and thereby to minimize distress. More recent iterations of this proposal have argued that dissociation occurs following severe traumatic events because the threat posed by traumatic experiences triggers a response in which the person needs to manage the distress in a way that permits compartmentalizing of their distress that permits them to cope with the adversity (Nijenhuis & van der Hart, 2011; van der Kolk & van der Hart, 1989). Theorists interested in acute reactions to trauma have argued that peritraumatic dissociative reactions will lead to subsequent PTSD because these reactions impede access to trauma memories, and thereby impair processing of these memories, which in turn leads to PTSD (Spiegel, Koopmen, Cardeña, & Classen, 1996). Consistent with this proposal is evidence that peritraumatic dissociation is linked to more severe traumatic experiences (Zatzick, Marmar, Weiss, & Metzler, 1994).

One factor that may contribute to the occurrence of peritraumatic dissociation is the extent to which a person dreads the occurrence of the traumatic event actually harming them. Whereas some events occur without warning (e.g., being hit by a truck from behind without any notice of the impending accident), others happen in a way that the person is fully aware of the imminent threat prior to it occurring (e.g., losing control of one's car and experiencing the fear as it falls down a cliff while the driver waits for the impact as it hits the ground beneath). It is possible that people who experience the dread of the imminent traumatic impact are more likely to experience peritraumatic dissociation than those who experience it without warning because they have greater opportunity to perceive the threat, and thereby engage in more dissociative responses. To understand the factors that contribute to peritraumatic dissociation more clearly, we investigated the extent to which peritraumatic dissociation in recently traumatized people was predicted by the extent they perceived warning of the impact of the traumatic event.

## Method

### Participants

Randomized injured hospital admissions to four level 1 trauma centers across Australia were recruited between January 2005 and February 2006. Patients who met entry criteria were randomly selected using an automated, random assignment procedure, stratified by length of stay. This approach was adopted so we did not differentially recruit patients who had longer hospital stays because they may be more accessible. The Research and Ethics Committee at each hospital approved the study. Inclusion criteria included: aged between 18 and 70 years of age; could understand and speak English proficiently; and had a hospital admission of greater than 24 hours following traumatic injury. Individuals were excluded from the study if they had any traumatic brain injury because of the potential confound between dissociative symptoms and traumatic brain injury (Bryant, 2001). Additional exclusion criteria included being currently psychotic or suicidal, non-Australian visitors, cognitively impaired, or under police guard. Following written informed consent, trained clinicians conducted a clinical interview and participants completed a self-report questionnaire booklet.

Five hundred participants were initially approached to complete the measures, and 445 (89%) agreed to participate and completed all the measures. Of these, 202 (45%) had a mild traumatic brain injury (MTBI) and were excluded from analysis on the basis that the frequent occurrence of transient loss of consciousness confounds their capacity to be aware of the warning of a traumatic event, and that symptoms associated with MTBI overlap markedly with dissociative responses (Bryant, 2001); accordingly, 243 participants were retained for analysis. The sample comprised 190 (78%) females and 53 (22%) males of mean age 38.50 years (SD=13.50) who had survived a motor vehicle accident (141, 58%), assault (14, 6%), traumatic fall (41, 17%), or other traumatic accident (47, 19%). In terms of the final sample, 97 (40%) were married, 143 (59%) had university or technical training, and 153 (63%) were in full-time employment.

### Procedure

Participants were approached within the trauma centers when medically stable (mean 4.04 days post injury, *SD=*17.59). Researchers conducted a structured interview concerning the injury, and in this interview they were asked the following question concerning the extent to which they perceived warning about the impact of the event occurring: *Some people have their injury happen totally out of the blue, some people have a few seconds warning, and some people have quite a bit of time before the injury finally happens to them. How much warning did you have of your injury?* Responses were coded as: no warning, 1–5 seconds, 6–10 seconds, 11–60 seconds, more than 60 seconds, or no memory of the extent of warning. Participants were also asked to complete a self-report booklet of questionnaires that contained the Peritraumatic Dissociative Experiences Questionnaire (PDEQ, Marmar, Weiss, & Metzler, 1997). The PDEQ is a self-report measure that comprises 10 items that are each scored on a five-point Likert scale. The PDEQ possesses two factors that index Impaired Awareness (i.e., alterations in perception) and Derealization (i.e., depersonalization and derealization) (Brooks et al., 2009). Whereas Impaired Awareness indexes factors that reflect narrowed attention during heightened arousal, Derealization indexes responses that involve altered experiences of oneself or one's environment.

Information regarding demographic, hospital admission, and injury-related factors were obtained from medical records and trauma registries from each of the hospitals. Injury information included the Injury Severity Score (American Association for Automotive Medicine, 1990), which is a measure of overall injury severity, cause of traumatic injury, hospitalization length, and presence of mild traumatic brain injury.

### Data analysis

To determine the relationship between degree of perceived warning and peritraumatic dissociation, we conducted two separate hierarchical linear regressions to predict PDEQ-Awareness and PDEQ-Derealization, respectively. Perceived warning score values were z-transformed to accommodate the non-linear pattern of warning scores. Predictors entered into the regression analyses were gender at Step 1 because of evidence that females are more likely to experience posttraumatic stress (Olff, Langeland, Draijer, & Gersons, 2007); Injury Severity Score at Step 2 because of evidence that dissociative responses are associated with more severe traumatic events (Zatzick et al., 1994); the type of trauma exposure (motor vehicle accident versus other types) at Step 3, and degree of perceived warning at Step 4 to test our prediction.

## Results

### Extent of perceived warning

In terms of the proportion of participants who reported different lengths of time until the impact occurred, 117 (48.1%) reported no warning, 115 (47.3%) reported warning of 1–5 seconds, three (1.2%) reported warning of 6–10 seconds, four (1.6%) reported warning of 11–60 seconds, and three (1.2%) reported not remembering how much warning they received.

The extent of perceived warning differed in terms of gender [female: 0.75 (SD=1.02), male: 0.57 (SD=0.72), *t* (600)=2.35, *p=*0.02], type of trauma [motor vehicle accidents: *M=*74, SD=86, other: *M=*0.47, SD=72, *t* (600)=4.04, *p=*0.001]. Perceived warning was positively associated with ISS (*r=*0.56, *p=*0.03).

### Relationship between extent of warning and peritraumatic dissociation

Table 1 presents the summary models of the linear regressions. The model predicting PDEQ-Awareness was significant, F (3, 236)=3.41, *p<*0.05. The only significant predictor of PDEQ-Awareness was female gender (*p<*0.001). The model predicting of PDEQ-Derealization was also significant, F (3, 236)=11.53, *p<*0.001. The significant predictors of PDEQ-Derealization were female gender (*p<*0.001) and degree of perceived warning (*p<*0.000).^1^

**Table 1:** Summary of hierarchical regression models

	PDEQ-Awareness				PDEQ-Derealization			
		
Step	Variable	B	SEB	ß	Variable	B	SE B	ß
Step 1	Gender	1.14	0.47	0.16*	Gender	1.54	0.37	0.26**
Step 2	ISS	0.04	0.03	0.09	ISS	–0.01	0.02	–0.05
Step 3	Trauma type	–0.09	0.40	–0.02	Trauma type	–0.04	0.32	–0.01
Step 4	Perceived warning	0.39	0.24	0.11	Perceived warning	0.38	0.19	0.20*

*Note*: PDEQ=Peritraumatic Dissociative Experiences Questionnaire. ISS=Injury Severity Score. Trauma type: motor vehicle accident=1, other traumas=2. Results for PDEQ-Awareness: Step 1 R2=0.02, ΔR2=0.02; Step 2 R2=0.03, ΔR2=0.01; Step 3 R2=0.03, ΔR2=0.00; Step 4 R2=0.04, ΔR2=0.1. Results for PDEQ-Derealization: Step 1 R2=0.07, ΔR2=0.07; Step 2 R2=0.7, ΔR2=0.00; Step 3 R2=0.07, ΔR2=0.00; Step 4 R2=0.09, ΔR2=0.02.

* *p<*0.05.

** *p<*0.001.

## Discussion

The current study provides initial evidence that peritraumatic dissociation is linked to the amount of time that trauma survivors perceive they had to anticipate the impact of the traumatic event. That is, the longer time one has to anticipate the outcome then the more one is likely to experience peritraumatic dissociation. Interestingly, this relationship was found for the Derealization subscale but not the Awareness subscale. Although the contribution of the extent of perceived warning to derealization was quite small relative to other predictive factors, the finding nonetheless provides insight into a mechanism that can explain this peritraumatic response. The Awareness factors reflect distortions in encoding of the experience, which often involve perceptual changes that are secondary to narrowed attention (Brooks et al., 2009). Narrowed attention is very common in the context of high arousal and is normally not associated with psychopathological responses (Kramer, Buckhout, & Eugenio, 1990; Sterlini & Bryant, 2002). In contrast, the Derealization subscale is more associated with distorted experiences of one's sense of self or environment. This dimension may be more associated with processes that distance oneself from the aversiveness of the experience. In related research, it has been suggested that adopting an observer perspective of one's memory of trauma is associated with higher levels of avoidance (Kenny & Bryant, 2007), and with evidence that the observer perspective of memory is linked to distressing emotions of the memory (McIsaac & Eich, 2004) and with subsequent PTSD (Kenny et al., 2009). This accords with evidence that the Derealization subscale correlates with severity of acute posttraumatic stress but the Awareness subscale does not (Brooks et al., 2009). It is possible that the more time one has to wait for the impact of the traumatic event to occur, the more aversive the anticipated experience is. This situation may lead to a stronger tendency to distance oneself from the experience because of the greater awareness of the potential dread of the impact.

It is also possible that greater arousal experienced as a result of the more drawn out anticipation of the traumatic event may contribute to more dissociative responses. Dissociative responses are often reported during extreme arousal, such as in panic attacks (Krystal, Woods, Hill, & Charney, 1991), and dissociative responses can be induced in recently trauma-exposed individuals with hyperventilation (Nixon & Bryant, 2006). Some theorists have posited that dissociation occurs when the person has a sense of loss of control, which exacerbates arousal (Collins, 2005). Consistent with this, elevated dissociation scores are associated with an external locus of control (Collins, & Ffrench, 1998). As trauma survivors experience a longer wait for the impact to occur, it is possible that they suffer more arousal and this leads to greater Derealization.

Interestingly, female gender predicted both the Derealization and Awareness subscale scores. This contrasts with a number of previous studies in which gender has not been found to predict peritraumatic dissociative responses (Koopman et al., 1994; Punamaki, Komproe, Qouta, Elmasri, & de Jong, 2005; Shalev et al., 1998). It is possible that the reason for this apparent discrepancy is the nature of population studied. Dissociative responses are more likely in severe and interpersonal traumatic events (Zatzick et al., 1994), and previous studies have comprised samples that are not identical to the current study. This discrepancy notwithstanding the current finding accords with much evidence that females are more likely to experience posttraumatic stress responses than males (Olff et al., 2007).

We recognize limitations to this study. One of the common peritraumatic dissociative experiences reported by trauma survivors is time distortion (Noyes & Kletti, 1977; Ursano et al., 1999). The general response is to describe time slowing as the person experiences the traumatic event. One theory of time estimation posits that aversive experiences may activate avoidance responses, which result in overestimates of time because of the person's motivation to avoid the perceived threat (Angrilli, Cherubini, Pavese, & Mantredini, 1997). This model has been supported by evidence that fear ratings are associated with overestimates of time (Campbell & Bryant, 2007). Consistent with this possibility, the association observed here may be attributed to participants who were more fearful believing more time had elapsed than had objectively transpired. Further, our conclusions are limited by the focus on survivors of traumatic injury; dissociation is more prevalent following severe trauma (Zatzick et al., 1994), and different patterns may be observed following a greater range of traumatic events, such as rape or torture. These limitations notwithstanding the current findings provide novel insight into one mechanism that leads to peritraumatic dissociative experiences. Although the DSM-5 definition of PTSD no longer recognizes peritraumatic responses as part of the criteria (Friedman, Resick, Bryant, & Brewin, 2011), these findings nonetheless point to one factor that may explain one of the key peritraumatic reactions that have been associated with subsequent PTSD.

## Footnotes

1 To eliminate the possibility of a confound between the time distortion item of the PDEQ and the estimate provided by participants of the time elapsed prior to the impact of the trauma, we re-analyzed the regression eliminating this item. This model predicting PDEQ-Derealization was significant, *F* (3, 236)=7.37, *p<*0.001. The significant predictors were female gender (*p<*0.001) and degree of warning (*p<*0.05).

## References

[CIT0001] American Association for Automotive Medicine (AAAM) (1990). The abbreviated injury scale 1990—revision.

[CIT0002] Angrilli A, Cherubini P, Pavese A, Mantredini S (1997). The influence of affective factors on time perception. Perception and Psychophysics.

[CIT0003] Breh D. C, Seidler G. H (2007). Is peritraumatic dissociation a risk factor for PTSD?. Journal of Trauma and Dissociation.

[CIT0004] Brooks R, Bryant R. A, Silove D, Creamer M, O'Donnell M, McFarlane A. C (2009). The latent structure of the peritraumatic dissociative experiences questionnaire. Journal of Traumatic Stress.

[CIT0005] Bryant R. A (2001). Posttraumatic stress disorder and traumatic brain injury: Can they co-exist?. Clin Psychol Rev.

[CIT0006] Bryant R. A (2007). Does dissociation further our understanding of PTSD?. Journal of Anxiety Disorders.

[CIT0007] Campbell L. A, Bryant R. A (2007). How time flies: A study of novice skydivers. Behaviour Research and Therapy.

[CIT0008] Cardeña E, Spiegel D (1993). Dissociative reactions to the San Francisco Bay Area earthquake of 1989. American Journal of Psychiatry.

[CIT0009] Collins F. E (2005). Dissociation in Australia. Journal of Trauma Practice.

[CIT0010] Collins F. E, Ffrench C. H (1998). Dissociation, coping strategies and locus of control in a non-clinical population: Clinical implications. Australian Journal of Clinical and Experimental Hypnosis.

[CIT0011] Ehlers A, Mayou R. A, Bryant B (1998). Psychological predictors of chronic posttraumatic stress disorder after motor vehicle accidents. Journal of Abnormal Psychology.

[CIT0012] Feinstein A (1989). Posttraumatic stress disorder: A descriptive study supporting DSM-III – R criteria. American Journal of Psychiatry.

[CIT0013] Friedman M. J, Resick P. A, Bryant R. A, Brewin C. R (2011). Considering PTSD for DSM-5. Depression and Anxiety.

[CIT0014] Harvey A. G, Bryant R. A (2002). Acute stress disorder: A synthesis and critique. Psychological Bulletin.

[CIT0015] Janet P (1907). The major symptoms of hysteria.

[CIT0016] Kenny L. M, Bryant R. A (2007). Keeping memories at an arm's length: Vantage point of trauma memories. Behaviour Research and Therapy.

[CIT0017] Kenny L. M, Bryant R. A, Silove D, Creamer M, O'Donnell M, McFarlane A. C (2009). Distant memories: A prospective study of vantage point of trauma memories. Psychological Science.

[CIT0018] Koopman C, Classen C, Spiegel D. A (1994). Predictors of posttraumatic stress symptoms among survivors of the Oakland/Berkeley firestorm. American Journal of Psychiatry.

[CIT0019] Kramer T, Buckhout R, Eugenio P (1990). Weapon focus, arousal, and eyewitness memory: Attention must be paid. Law and Human Behavior.

[CIT0020] Krystal J. H, Woods S. W, Hill C. L, Charney D. S (1991). Characteristics of panic attack subtypes: Assessment of spontaneous panic, situational panic, sleep panic, and limited symptom attacks. Comprehensive Psychiatry.

[CIT0021] Marmar C. R, Weiss D. S, Metzler T. J, Wilson J. P, Keane T. M (1997). The peritraumatic dissociative experiences questionnaire. Assessing psychological trauma and PTSD.

[CIT0022] McIsaac H. K, Eich E (2004). Vantage point in traumatic memory. Psychological Science.

[CIT0023] Murray J, Ehlers A, Mayou R. A (2002). Dissociation and post-traumatic stress disorder: Two prospective studies of road traffic accident survivors. British Journal of Psychiatry.

[CIT0024] Nijenhuis E. R, van der Hart O (2011). Dissociation in trauma: A new definition and comparison with previous formulations. Review] Journal of Trauma and Dissociation.

[CIT0025] Nixon R. D. V, Bryant R. A (2006). Dissociation in acute stress disorder after a hyperventilation provocation test. Behavioural and Cognitive Psychotherapy.

[CIT0026] Noyes R, Kletti R (1977). Depersonilization in response to life-threatening danger. Comprehensive Psychiatry.

[CIT0027] Olff M, Langeland W, Draijer N, Gersons B. P. R (2007). Gender differences in posttraumatic stress disorder. Psychological Bulletin.

[CIT0028] Punamaki R. L, Komproe I. H, Qouta S, Elmasri M, de Jong J (2005). The role of peritraumatic dissociation and gender in the association between trauma and mental health in a Palestinian community sample. American Journal of Psychiatry.

[CIT0029] Shalev A. Y, Freedman S, Peri T, Brandes D, Sahar T (1997). Predicting PTSD in trauma survivors: Prospective evaluation of self-report and clinician-administered instruments. British Journal of Psychiatry.

[CIT0030] Shalev A. Y, Freedman A, Peri T, Brandes D, Sahara T, Orr S (1998). Prospective study of posttraumatic stress disorder and depression following trauma. American Journal of Psychiatry.

[CIT0031] Spiegel D, Koopmen C, Cardeña C, Classen C, Michelson L. K, Ray W. J (1996). Dissociative symptoms in the diagnosis of acute stress disorder. Handbook of dissociation.

[CIT0032] Sterlini G. L, Bryant R. A (2002). Hyperarousal and dissociation: A study of novice skydivers. Behaviour Research and Therapy.

[CIT0033] Ursano R. J, Fullerton C. S, Epstein R. S, Crowley B, Vance K, Kao T.-C (1999). Peritraumatic dissociation and posttraumatic stress disorder following motor vehicle accidents. American Journal of Psychiatry.

[CIT0034] van der Kolk B, van der Hart O (1989). Pierre Janet and the breakdown of adaptation in psychological trauma. American Journal of Psychiatry.

[CIT0035] Velden P. G. v. d, Kleber R. J, Christiaanse B, Gersons B. P. R, Marcelissen F. G. H, Drogendijk A. N (2006). The independent predictive value of peritraumatic dissociation for postdisaster intrusions, avoidance reactions, and PTSD symptom severity: A 4-year prospective study. Journal of Traumatic Stress.

[CIT0036] Zatzick D. F, Marmar C. R, Weiss D. S, Metzler T (1994). Does trauma-linked dissociation vary across ethnic groups?. Journal of Nervous and Mental Disease.

